# Tumor Acidity as Evolutionary Spite

**DOI:** 10.3390/cancers3010408

**Published:** 2011-01-20

**Authors:** Khalid O. Alfarouk, Abdel Khalig Muddathir, Mohammed E. A. Shayoub

**Affiliations:** 1 Department of Biotechnology, Africa City of Technology, Khartoum, Sudan; 2 Department of Pharmaceutics, Faculty of Pharmacy, University of Khartoum, Khartoum, Sudan; E-Mail: shayoub2004@hotmail.com; 3 Department of Pharmacognosy, Faculty of Pharmacy, University of Khartoum, Khartoum, Sudan; E-Mail: muak46@yahoo.com

**Keywords:** Warburg-effect, cannibalsim, spite, MAICS

## Abstract

Most cancer cells shift their metabolic pathway from a metabolism reflecting the Pasteur-effect into one reflecting the Warburg-effect. This shift creates an acidic microenvironment around the tumor and becomes the driving force for a positive carcinogenesis feedback loop. As a consequence of tumor acidity, the tumor microenvironment encourages a selection of certain cell phenotypes that are able to survive in this caustic environment to the detriment of other cell types. This selection can be described by a process which can be modeled upon spite: the tumor cells reduce their own fitness by making an acidic environment, but this reduces the fitness of their competitors to an even greater extent. Moreover, the environment is an important dimension that further drives this spite process. Thus, diminishing the selective environment most probably interferes with the spite process. Such interference has been recently utilized in cancer treatment.

## 1. Cancer Metabolism Creates an Acidic Environment

Both cancer and normal cells depend mainly on glucose metabolism to generate ATP to carry out normal maintenance and proliferation, where the initial steps of glucose metabolism result in the formation of pyruvate and generate two moles of ATP per mole of glucose. Switching from aerobic to anaerobic metabolism in the absence of oxygen is termed the Pasteur-effect [1]. Alternatively, under anaerobic conditions, pyruvate is converted to lactic acid. This is typically observed clinically in skeletal muscles during severe exercise where oxygen concentrations are exhausted and glucose metabolism is limited to the conversion to lactic acid. Nearly 100 years ago, Otto Warburg first observed that cancer cells metabolize glucose to lactate even in presence of oxygen—an observation that has been repeatedly confirmed and is now termed the Warburg effect [2]. Since the end product is lactic acid and because the reduced efficiency in ATP conversion requires increased glucose flux, the up-regulation of glycolysis in normal or cancer cells creates an acidic interstitial/extracellular environment [3].

These observations create a conundrum. It is widely assumed that the malignant phenotype arises through a process that is formally analogous to Darwinian evolution. Thus, if cancer results from prolonged “somatic evolution”, then any common phenotypic property observed in a malignant cell must confer a fitness advantage. On the contrary, aerobic glycolysis would confer two significant proliferative disadvantages: (1) It is significantly (18-fold!) less efficient than oxidative metabolism in producing energy, thus requiring far greater glucose uptake and use; and, (2) It produces a significantly acidic microenvironment that is toxic to mammalian cells. When combined with hypoxia, this metabolic microenvironment becomes quite caustic.

**Figure 1.**
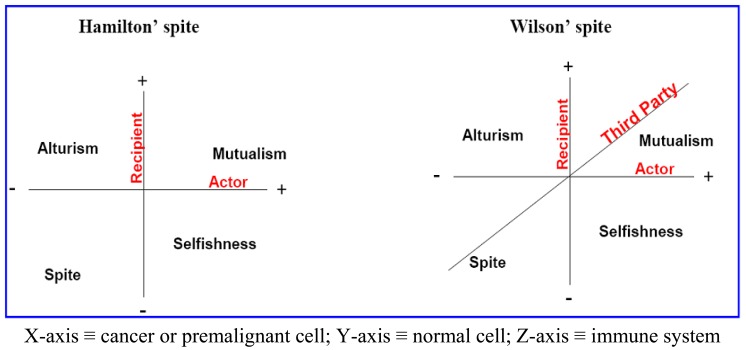
A comparison between the interaction spaces presented by the spite models proposed by Hamilton (left panel) and Wilson (right panel). **Mutualism** could be represented through the support of tumor growth by nonmalignant cells [30]. Moreover, some cancer cells become addicted to InterLeukin-3 to survive (primed cell for death) and so benefit from the immune system, and this is a kind of **selfishness** [31]. Yet, microenvironment acidity-induced cancer spite (MAICS) results in excessive cell death and eventually might prevent its spreadability and so it lies under the altruism umbrella because it results in an encapsulated tumor [32]. Cannibalism at the end results in death of both normal and malignant cells under the context of organismal selection so it is compatible with **spite**. Therefore, it would be very interesting if further studies carry on for determining how tumors handle the thresholds of the four quadrants.

**Figure 2.**
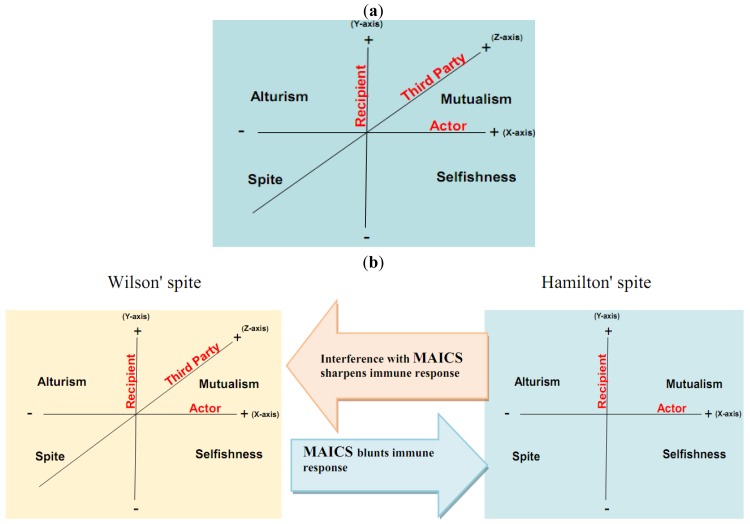
Here we propose a hypothetical model in which the environment where the player interactions are carried out (*i.e.*, the acidic extracellular space; blue background) can play an important role in determining the choice and dynamics of spite. At this point, it is not possible to determine if there is a space-time quadrant where the environment has a greater or lesser effect and this will be an interesting point for future research. (**a**) Because microenvironment acidity-induced cancer spite (MAICS) blunts the immune system [33], interference with MAICS would probably create a shifting to Wilsonian spite. Thus, the third party reappears (Z-axis in (b) (Wilsonian spite) (**b**) This kind of shifting does not misconstrue to malignant and normal cells only but a spite process could happen also between secondary tumor cells (cannibal cells) and primary tumor cells.

Here, we propose that aerobic glycolysis confers an evolutionary advantage due to an adaptive strategy that is commonly termed “spite”. In brief, an organism can evolve a less fit phenotype only if it alters the local adaptive landscape in such a way that it reduces the fitness of all competing populations even more. So, cancer cells, as actors, increase their fitness compared to normal cells as recipients. The acidic environment will select for acid-adapted phenotypes not the glycolytic phenotype. Only after this adaptation will the glycolytic phenotype confer an advantage. This combination of phenotypes then confers an advantage because it increases extracellular matrix (ECM) degradation to facilitate invasion, induces death in normal cells, increases vascular endothelial growth factor (VEGF) release, and reduces the effectiveness of cytotoxic T cells in generating an immune response to tumor antigens. In this general theory, there has developed two types of spites: (i) Hamiltonian spite and (ii) Wilsonian spite, which is a modified Hamiltonian spite because he added a third party that gains benefit or costs the interaction of actor and recipient [4] that could be represented by the immune system in our model (z-axis) (see Figure 1). Here, we propose the environment as an additional fourth dimension because spite might not occur in the absence of a suitable environment (see Figure 2a).

## 2. Consequences of Microenvironment Acidity

It seems that carcinogenesis can undergo a positive feedback mechanism (control) and/or propagation reaction where once acidity appears around the cell, the tumor microenvironment become hostile, which could be seen as a point of no return. This acidic microenvironment selects pre-malignant (actor) glycolytic traits that adapt in the acidic microenvironment with normal cells (recipient) and, in this way; the acidic microenvironment produces Hamiltonian spite (Selfishness).

In a positive feed-back cycle, these glycolytic traits then aggravate the microenvironment acidity that selectively increases the malignant phenotype. This hostile microenvironment selects more virulent types of cells expressing invasion promoting traits such as degradation of ECM, activation of VEGF, carbonic anhydrase [5,6], lactate dehydrogenase, cathepsin B and matrix metalloproteinases MMP-2 and MMP-9 [7,8]. Moreover, the acidic microenvironment inhibits the immune response [9]. Finally, the acidic microenvironment generates extremely virulent cell types that can phagocytosize stroma, normal cells, sibling cancer cells, yeast, *etc.*, in a process called Cannibalism [10]. Cannibalism is a characteristic of secondary tumors, not primary tumors, and it occurs when malignant cells face starvation (low nutrients level), *i.e.*, shifting from the Warburg-effect into Cannibalism. Cannibalism, as an indicator of reverse evolution of cancer [11-13], represents an additional tool of spite by increasing fitness; under starvation conditions cancer cells acquire the life style to survive, normal cells die. Thus, tumor cannibalism is a second tool of spite when the Warburg-effect does not reconcile with this starved environment. We do not know if tumor dormancy could represent an additional spite model or not if we compare it with normal cells under a high caustic environment due to tumor acidity or chemotherapy. Because metastases is an efficient process [14], tumor spite represents a successful strategy for tumor survival.

The interaction of the cannibal cell (actor) alone with the recipient cell could be described as an example of Hamiltonian spite (selfishness). Interestingly, this Hamiltonian spite could be expanded to Wilsonian spite, where the primary tumor (third party) obtains additional beneficial effects from such a suppression of immune cells. Hence, tumor acidity might represent an attractive model for mixed types of spite. In this way, alteration of the acidic microenvironment eventually results in catastrophic dismantling carcinogenesis cascade (Gatenby-Gillies' Model) [15] via a positive feedback loop that finally results in spite. We suggest the term “microenvironment acidity-induced cancer spite (MAICS)” to define this acidic microenvironment-driven spite cascade (see Figure 2b).

## 3. Interference with MAICS (Anti-MAICS)

Recently, the targeting of MAICS (microenvironment acidity-induced cancer spite) has been suggested to be an attractive strategy in the war against cancer [16] that could most probably be achieved by the simultaneous use of several chemotherapeutic agents including: Proton Pump Inhibitors (PPIs) [17,18], bicarbonate (HCO_3_^-^), carbonic anhydrase inhibitors e.g., Acetazolamide [19-21], Na^+^-H^+^ exchanger inhibitors e.g., Amiloride [22-24], H^+^- ATP Synthase inhibitors e.g., Resveratrol [25-27]. Furthermore, spite represents a successful strategy in the generation of resistance [28], so once again MAICS would be an attractive target against chemotherapeutic resistance [29]

## 4. Conclusions

Our hypothesis is that the Warburg effect and tumor cannibalism are evolutionary consequences of a process termed ‘spite’, which arises through a series of steps in carcinogenesis. Thus, the tumor microenvironment is a suitable medium created to carry out these steps that provide tumor cells fitness and, in this context, targeting the tumor microenvironment represents a novel and potentially useful strategy in cancer therapy.
